# Caveolin-1 Influences Vascular Protease Activity and Is a Potential Stabilizing Factor in Human Atherosclerotic Disease

**DOI:** 10.1371/journal.pone.0002612

**Published:** 2008-07-02

**Authors:** Juan A. Rodriguez-Feo, Willem E. Hellings, Frans L. Moll, Jean-Paul P. M. De Vries, Ben J. van Middelaar, Ale Algra, Joost Sluijter, Evelyn Velema, Theo van der Broek, William C. Sessa, Dominique P. V. De Kleijn, Gerard Pasterkamp

**Affiliations:** 1 Department of Cardiology, Experimental Cardiology Laboratory, University Medical Center, Utrecht, The Netherlands; 2 Department of Vascular Surgery, University Medical Center, Utrecht, The Netherlands; 3 Department of Vascular Surgery, St. Antonius Hospital, Nieuwegein, The Netherlands; 4 Julius Center for Health Sciences and Primary Care, University Medical Center, Utrecht, The Netherlands; 5 Department of Neurology, Rudolf Magnus Institute, UMC Utrecht, Utrecht, The Netherlands; 6 Department of Pharmacology, Boyer Center for Molecular Medicine, Yale University School of Medicine, New Haven, Connecticut, United States of America; 7 Interuniversity Cardiology Institute of the Netherlands, Utrecht, The Netherlands; Monash University, Australia

## Abstract

Caveolin-1 (Cav-1) is a regulatory protein of the arterial wall, but its role in human atherosclerosis remains unknown. We have studied the relationships between Cav-1 abundance, atherosclerotic plaque characteristics and clinical manisfestations of atherosclerotic disease.We determined Cav-1 expression by western blotting in atherosclerotic plaques harvested from 378 subjects that underwent carotid endarterectomy. Cav-1 levels were significantly lower in carotid plaques than non-atherosclerotic vascular specimens. Low Cav-1 expression was associated with features of plaque instability such as large lipid core, thrombus formation, macrophage infiltration, high IL-6, IL-8 levels and elevated MMP-9 activity. Clinically, a down-regulation of Cav-1 was observed in plaques obtained from men, patients with a history of myocardial infarction and restenotic lesions. Cav-1 levels above the median were associated with absence of new vascular events within 30 days after surgery [0% vs. 4%] and a trend towards lower incidence of new cardiovascular events during longer follow-up. Consistent with these clinical data, Cav-1 null mice revealed elevated intimal hyperplasia response following arterial injury that was significantly attenuated after MMP inhibition. Recombinant peptides mimicking Cav-1 scaffolding domain (Cavtratin) reduced gelatinase activity in cultured porcine arteries and impaired MMP-9 activity and COX-2 in LPS-challenged macrophages. Administration of Cavtratin strongly impaired flow-induced expansive remodeling in mice.This is the first study that identifies Cav-1 as a novel potential stabilizing factor in human atherosclerosis. Our findings support the hypothesis that local down-regulation of Cav-1 in atherosclerotic lesions contributes to plaque formation and/or instability accelerating the occurrence of adverse clinical outcomes. Therefore, given the large number of patients studied, we believe that Cav-1 may be considered as a novel target in the prevention of human atherosclerotic disease and the loss of Cav-1 may be a novel biomarker of vulnerable plaque with prognostic value.

## Introduction

Atherosclerotic plaque formation, destabilization and rupture with subsequent thrombus formation give rise to acute coronary syndromes [1], symptomatic carotid artery disease [2] and sudden cardiac death [3]. Plaque rupture is associated to an elevated inflammatory response [4], increased proteolytic activity [5]; [6] and intra-plaque bleeding [7]. The current lack of knowledge about the natural history of human atherosclerotic disease and plaque progression restricts the possibility to identify subjects at risk for cardiovascular events. In order to evaluate the relationship between local protein expression in atherosclerotic plaques and future vascular events, the ATHERO-EXPRESS vascular bio-bank was established [8] . The main goal of this study is to identify the expression patterns of specific proteins expressed within the vascular tree that may make patients prone to suffer cardiovascular events in all vascular territories. These proteins, diffusely expressed in the vasculature, could then be locally detected using local molecular imaging or in endarterectomy specimens, and serve as a surrogate marker to identify the patient at risk for future adverse cardiovascular events, the so-called vulnerable patient [9]. In this context, we have tested the possibility that Caveolin-1 (Cav-1) plaque abundance is related to plaque and patient stability.

Cav-1 is the main coat protein of caveolae and is expressed by different vascular cells [10]. Caveolae and Cav-1 have emerged as novel targets in the control of various important cellular processes involved in the maintenance of cardiovascular homeostasis such as protein trafficking, lipid metabolism and signal transduction [11]. The involvement of Cav-1 in arterial occlusive disease like atherosclerosis still remains controversial and not well understood [12]. Cav-1/ApoE double-knockout mice show less lipid accumulation in the aorta, while the absence of Cav-1 promotes smooth muscle cells proliferation increasing intimal hyperplasia upon carotid injury [13]; [14]. In addition, the analysis of plaques from hypercholesterolemic rabbits and humans showed reduced Cav-1 levels, suggesting athero-protective actions for Cav-1 [15]; [16]. However; the study of the relationships between Cav-1 abundance, atherosclerotic plaque phenotype and clinical manifestations of atherosclerotic disease has not been undertaken yet.

Therefore, we determined Cav-1 expression in carotid atherosclerotic plaques from a cohort of 378 patients undergoing carotid endarterectomy obtained from the ATHERO-EXPRESS study. We hypothesized that plaque levels of Cav-1 might be related to plaque morphology, inflammation and matrix metalloprotease (MMP) activity. We also investigated if local Cav-1 expression levels in the carotid plaque were related to clinical characteristics. In addition, the design of the bio-bank study allowed the execution of a follow up with the objective to study the predictive value of local Cav-1 expression for the future development of cardiovascular adverse events and thus might help identifying patients at risk. Additionally, we hypothesized that potential associations of Cav-1 expression with adverse outcomes could be explained by an inhibitory effect on MMP activity in the atherosclerotic lesion. In order to address this hypothesis we further analyzed the effect of Cav-1 on intimal hyperplasia in Cav-1 null mice and whether an increased intimal hyperplasia response could be attenuated by MMP blockade. In addition, we studied the impact of Cav-1 scaffolding domain (CSD) on gelatinase activity, COX-2 expression and expansive arterial remodeling in vitro and in mice.

This clinical and pre- clinical data provides evidence supporting an important role for Cav-1, and its related peptides, in vascular pathologies such as intimal hyperplasia, expansive remodeling and human atherosclerotic plaque destabilization and rupture.

## Methods

### Human study

#### The Athero-Express vascular bio-bank

Athero-Express is an ongoing vascular bio-bank project with the goal to investigate locally expressed plaque markers in relation to clinical presentation and clinical outcome [8]. The bio-bank project is running in two Dutch hospitals and was approved by the ethical commitees of the Antonius hospital Nieuwegein, The Netherlands and the UMCU, Utrecht, the Netherlands. Written informed consent is obtained from all patients. Criteria for patient selection for carotid endarterectomy (CEA) were based upon the recommendation of NASCET and ESCT for symptomatic patients and ASCT for asymptomatic patients [17]; [18]. Stenosis degree was assessed by duplex. At baseline, medication use, cardiovascular risk factors, history of cardiovascular disease, and other baseline characteristics were retrieved from questionnaires. Additional clinical data were recorded from patient charts. Lipid spectra and hs-CRP were measured in blood samples drawn at baseline. For the current study, carotid endarterectomy patients between the start of the study in April 2002 until April 2006 were included.

#### Follow-up Protocol and Clinical Outcome Events

After carotid surgery, patients were followed yearly up to 3 years (mean: 23 months). The primary outcome was defined as vascular event: the composite of vascular death, non-fatal myocardial infarction, non-fatal stroke, non-fatal rupture of an abdominal aortic aneurysm, and vascular surgical intervention, whichever occurred first. Additional outcomes were: 1) myocardial infarction (fatal and non-fatal) and coronary revascularization and 2) ischemic stroke. Definitions and assessment procedures of the outcome events were described previously [19].

#### Tissue Sampling

Carotid endarterectomy was performed by an open, non-eversion technique with careful dissection of the atherosclerotic plaque. Following excision, the plaque was immediately transferred to the laboratory to undergo standardized processing. First, it was divided into 5mm cross-sectional segments. The culprit lesion, defined as the segment with greatest plaque burden, was fixated in 4% formalin for 7 days and then decalcified in EDTA and embedded in paraffin. The other segments were snap frozen in liquid nitrogen and stored at −80°C. Protein extraction was performed on the carotid segments adjacent to the culprit lesion by mechanical crushing followed by 1) protein isolation with TriPure reagent, according to the manufacturer's protocol (Boehringer Mannheim, Germany) and 2) by dissolving in 40 mM Tris-HCl (pH = 7.5) at 4°C. Segments of macroscopically non-atherosclerotic mammary arteries (n = 9) obtained during coronary artery bypass surgery served as a non-diseased control.

#### Histological Assessment of Carotid Atherosclerotic Plaques

All plaques were characterized as described earlier [8]. The following stainings were applied on serial cross-sections (5 µm) of paraffin embedded tissue and semi-quantitatively analyzed as no, minor, moderate or heavy staining by observers blinded for patient characteristics: macrophages (CD68), smooth muscle cells (alpha-actin), collagen (picro-sirius red), calcifications (HE) and thrombus (HE and picro-sirius red). The size of the lipid core was visually estimated as a percentage of plaque area using H&E and picro-sirius stainings (<10%, 10%–40%, >40%). In addition, computerized measurements of CD68 and alpha-actin were performed to assess macrophage and smooth muscle content respectively. Representative images of semi-quantitative staining can be found in [20].

#### Determination of Intra-plaque Interleukin levels, MMP activity and EMMPRIN levels

Interleukin-6 and -8 (IL-6 and IL-8) concentrations were determined with a multiplex suspension array system according to the manufacturer's protocol (Bio-Rad Laboratories, Hercules, CA). MMP-2 and MMP-9 activities and Extracellular Matrix Metalloproteinase Inducer (EMMPRIN) levels were measured in a randomly selected subgroup of 128 patients. MMP-2 and MMP-9 activity measurements were performed with Biotrak activity assays RPN 2631 and RPN 2634, respectively (Amersham Biosciences, Buckinghamshire, UK). EMMPRIN expression levels were determined by Western blotting as described previously [21]. The ratio between the two forms of EMMPRIN (58 KD highly glycosylated and a 45 KD less glycosylated forms) was calculated.

#### Cav-1 Immunohistochemistry

Serial cross-sections (5 µm) from carotid endarterectomy specimens and mammary arteries were deparaffinized and rehydrated, boiled in sodium citrate and blocked in 10% normal goat serum. The sections were incubated for 1 hour at room temperature with 0.2 µg/ml polyclonal rabbit-anti human-Cav-1 antibody (610059, BD biosciences, Franklin Lakes, NJ), as determined by titration, followed by biotinylated goat-anti-rabbit antibody (Vector, Burlingame, CA) and horseradish peroxidase (HRP) labeled streptavidin (Vector). Staining was developed with AEC substrate with Mayer's haemotoxylin as counterstaining. Negative controls were obtained avoiding the primary antibody. Double labeling for Cav-1 and alpha-actin SMC (Sigma,St Louis) and CD34 (Dako, Denmark) were also performed.

### Animal Studies

Animals were housed conformed to the Guide for the care and use of Laboratory Animals (NIH publication No.85-23, 1985) and all experiments were approved by the ethical committee on animal experiments of the University Medical Center, Utrecht. BALB/c mice, Cav-1 null mice (Cav-1^tm1Mls^) and appropriate Wild type (WT) genetic background controls were purchased by the Jackson laboratories (Bar Harbor, MA).

#### Induction of Intimal Hyperplasia and Matrix- Metalloprotease (MMP) inhibition

A group of Cav-1 null mice were treated daily with doxycycline (DOX), an orally available MMP inhibitor, in drinking water at the dose of 30 mg/kg/day as used earlier [22] .The treatment started a week before cuff placement and was continued for another 3 weeks. Polyethylene cuffs, to induce intimal hyperplasia, were placed around the right femoral artery in WT (n = 11), Cav-1 null (n = 12) and Cav-1 null+DOX (n = 11) mice. The animals were sacrificed 3 weeks after cuff placement and the femoral arteries were harvested and analyzed as reported before [23] .

#### Recombinant Peptides Experiments

Synthetic peptides corresponding to scaffolding domain of human Caveolin-1 (residues 82–101) (Cavtratin) and scrambled version were prepared as previously reported [24]; [25] . For *in vitro* experiments, final concentration of each peptide was achieved by diluting the stock in culture medium. For *in vivo* experiments, peptides were dissolved in 30% dimethyl sulfoxide (DMSO) solution in 0.9% saline buffer (Braun) and mini-osmotic pumps (Alzet) with an internal volume of 200 µl and infusion rate of 0.5 µL/ hour were filled with a daily dose of 1.5 mg/kg of Cavtratin or scrambled peptides. Subsequently, mini-osmotic pumps were attached to a small flexible catheter (Alzet). Catheters were surgically connected to the jugular vein of BALB/c mice and pumps were placed subcutaneously. Thereafter, a right carotid ligation was performed as a model for expansive remodeling in BALB/c (n = 14), BALB/c+scrambled (n = 6) and BALB/c+Cavtratin (n = 6) as described earlier [26]. Non-ligated arteries (BALB/c, n = 10) were used as a reference (0 days). After 4 weeks, the contralateral left carotids were pressure-fixed, harvested and embedded in paraffin. Total circumference area (EEL area), intimal and luminal area were measured after elastin staining and compared to non-ligated carotids (0 days). Expansive remodeling was defined as an increase in EEL area compared with the reference group. Data were analyzed by ANOVA.

#### Gelatin and in situ Zymography

Gelatin zymography was performed as described previously [27]. For in situ zymography, porcine arterial rings were embedded in Tissue-Tek (Sakura), sectioned (5 µm) and incubated at 37°C overnight with a fluorogenic gelatin substrate (DQ gelatin, Molecular Probes) to a final concentration of 25 µg/mL. Proteolytic activity was detected under the microscope as green fluorescence at 530 nm.

#### Culture of Porcine Arterial Rings

Internal mammary arteries were surgically harvested from 2 adult male pigs and sliced in approximately 0.5 cm rings. After washing with PBS, arterial rings were immediately frozen (0 days) and the remaining fragments were incubated overnight in presence or absence of Cavtratin or scrambled peptides in a serum-free D-MEM (Gibco) and after extensive washing were cultured in 5% FBS D-MEM for 3 days. Thereafter, samples were processed for gelatin and *in situ* zymography.

#### Human Cav-1, murine EMMPRIN and COX-2 Western Blotting

Equal amounts of total protein were denaturized and subjected to a SDS-PAGE in 10% or 12% polyacrylamide gels. Proteins were transferred onto nitrocellulose membranes (Schleier & Schuell, Dasel, Germany) and correct transfer was checked by Ponceau Red S staining. The membranes were incubated with either polyclonal rabbit-anti-human Cav-1 antibody (0.1 µg/mL, BD biosciences, Franklin Lakes, NJ), polyclonal goat anti EMMPRIN (0.4 µg/mL , G-19 Santa Cruz, Biotechnology) or polyclonal rabbit anti murine COX-2 (0.5 µg/mL, Cayman Chemicals, Ann Arbor, Mi) followed by incubation with appropriate HRP conjugated secondary antibodies. Signal detection was performed by enhanced chemiluminescence (Sigma, Saint Louis, MO). For western blotting in CEA samples, a pooled sample of mammary arteries (n = 6) was loaded on each gel as a positive control. Accordingly, in every gel, expression levels of Cav-1 in pooled mammary arteries were considered as 100. Cav-1 expression levels in non-atherosclerotic mammary arteries (n = 3) and CEA samples were standardized and calculated as percentages relative to standard positive control.

#### Cell Culture

Raw-264.7 murine macrophages were obtained from ATCC (Manassas, VA) and grown and propagated accordingly to manufacture's recommendations.

#### Data Analysis

Comparison of Cav-1 expression levels between different artery types and different patient groups was done by Mann Whitney U tests. The Mann Whitney U test was also used to test the association between Cav-1 measurements and semi-quantitatively measured plaque characteristics, comparing no and minor staining to moderate and heavy staining. For survival analysis, Cav-1 levels were dichotomized at the median. The group with low Cav-1 expression levels (<median) was compared with the group high Cav-1 measurements (> = median) by Kaplan-Meier survival analysis. Cox Proportional Hazard analysis was used to compute Hazard ratios (HR) with 95% confidence intervals [CI] and to adjust for sex, gender and plaque overall phenotype. Data from animal, ex vivo and cultured cells were analyzed by Mann-Whitney U and ANOVA tests. P-values <0.05 were considered statistically significant.

## Results

Baseline characteristics of the patient population are depicted in Table 1. In total 378 patients were included in this report, with a mean age of 67.4+/−8.8 years.

**Table 1:** Baseline characteristics of the studied population.

	n (%)
patient number	378
age, years (sd)	67.4 (8.8)
male	258 (68%)
hypertension	247 (70%)
diabetes	70 (20%)
smoking	88 (26%)
body mass index, kg/m^2^ (sd)	26.7 (4.5)
total cholesterol, mmol/l	5.0 (1.1)
HDL cholesterol, mmol/l	1.2 (0.37)
LDL cholesterol, mmol/l	3.0 (1.0)
triglycerides, mmol/l	2.1 (1.1)
hs-CRP, mg/l (IQR)	3.4 (1.6–6.9)
prior ipsilateral CEA	12 (3%)
prior vascular intervention	139 (35%)
history of myocardial infarction	72 (20%)
carotid stenosis grade	
50–64%	12 (3%)
65–89%	133 (35%)
90–99%	232 (62%)
symptoms	
asymptomatic	87 (23%)
ocular symptoms	51 (14%)
TIA	130 (35%)
stroke	102 (28%)

### Cav-1 expression pattern in Human Atherosclerotic Lesions

**Figure 1:**
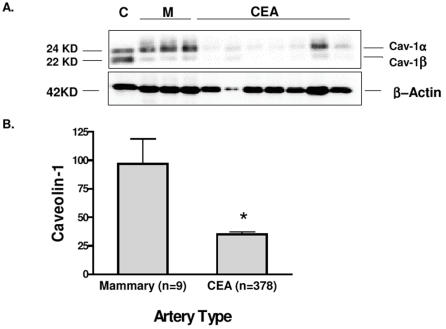
Cav-1 expression pattern in normal and atherosclerotic lesions. A: representative Western Blot. “C” denotes control (pooled sample), “M” denotes mammary arteries, “CEA” denotes carotid plaques. Detection of Cav-1 α and β isoforms (22 and 24 KD; above) and β-actin (42 KD; below) are shown. B: Quantification of Cav-1protein levels in carotid artery vs. control mammary arteries (mean and SE). * p = 0.001. Note that β−actin and Cav-1 expression patterns differ completely, indicating the specificity of Cav-1 down-regulation in CEA samples.

Cav-1 antibody was able to detect two bands with a relative molecular weight of 24KD and 22 KD demonstrating the presence of the two known isoforms of Cav-1 (α and β); (Figure 1A) Cav-1 protein expression levels were strongly reduced in atherosclerotic plaques compared with non-atherosclerotic mammary arteries (Figure 1B). The carotid plaques showed 64% lower expression levels compared to mammary arteries (95% CI: [31%–96%]) (Figure 1B).

**Figure 2:**
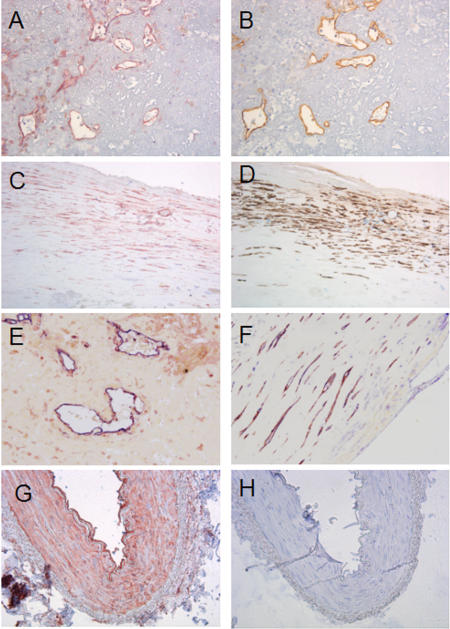
Cav-1 immunohistochemistry. A, C: Cav-1 staining on carotid plaques (red, 200x magnification). B: Endothelial staining (CD34, brown) on a consecutive section of A showing co-localization of Cav-1 and endothelium. D: Alpha-actin smooth muscle cell staining (brown) on a consecutive section of C, showing co-localization of Cav-1 and smooth muscle cells. E: Double-staining of Cav-1(red) and CD34 (blue),( 200X magnification). F: Double staining of Cav-1 (blue) and Alpha-actin smooth muscle cell sataining (red) ( 200X magnification). G: Cav-1 staining on a mammary artery, showing staining throughout the intima, media and adventitia. (100x magnification). H: Negative control of Cav-1 staining in a consecutive section of G, avoiding the primary antibody. Sections were counterstained with haemotoxylin except the double-stained sections (E and F) .

By immunohistochemistry, we found that both mammary arteries and human atherosclerotic lesions expressed Cav-1 (Figure 2). In mammary arteries, Cav-1 staining was distributed throughout the intimal, medial and adventitial layers. In carotid plaques, Cav-1 was frequently found in smooth muscle cells, the endothelium of neovascularized areas and endothelium aligning the vessel lumen. Co-localization with macrophages was not observed. No staining was observed in non-immune controls (Figure 2).

### Cav-1 Abundance and Plaque Characteristics

We further examined the relationships between Cav-1 expression levels and different plaque characteristics. Plaques with an atheromatous phenotype showed lower Cav-1 expression levels compared with fibrous plaques (p<0.001; Table 2). In addition, Cav-1 levels were significantly lowered in plaques with unstable characteristics such as high macrophage staining (p = 0.04), low smooth muscle cell staining (p<0.001), low collagen staining (p = 0.02), and high amount of thrombus (p = 0.005). There was no association between Cav-1 expression levels and extent of calcifications.

**Table 2:** Relationships between plaque histology and Cav-1 protein expression levels.

	Cav-1 levels				p-value
	***fibrous***	***fibro-atheromatous***	***atheromatous***		
***overall phenotype***	26.2 (11.7–53.3)	25.0 (7.7–56.0)	13.3 (4.5–32.9)		<0.001
	***no staining***	***minor staining***	***moderate staining***	***heavy staining***	
***macrophage staining***	24.0 (5.7–54.4)	23.0 (10.3–51.2)	20.0 (5.7–47.4)	14.5 (3.6–30.7)	0.04
***smooth muscle cell staining***	5.7 (2.3–27.4)	11.9 (3.5–25.4)	19.6 (6.4–45.7)	34.4 (18.2–71.2)	<0.001
***collagen staining***		15.6 (3.7–33.8)	20.4 (6.7–47.9)	26.1 (12.0–49.9)	0.02
***calcifications***	23.5 (5.9–50.1)	18.4 (4.9–35.7)	15.9 (7.4–41.2)	26.2 (7.7–55.7)	0.57
***thrombus***	30.1 (13.0–53.3)	17.4 (5.7–46.0)	14.6 (3.5–33.1)	10.7 (2.5–27)	0.005

The values given are the median Cav-1 levels and interquartile range in the respective staining group. P-values were calculated comparing Cav-1 expression levels between no and minor staining vs. moderate and heavy staining, and in case of overall phenotype: fibrous vs. atheromatous.

### Cav-1 Abundance, Proteolytic Activity and Inflammation-Human data

**Figure 3:**
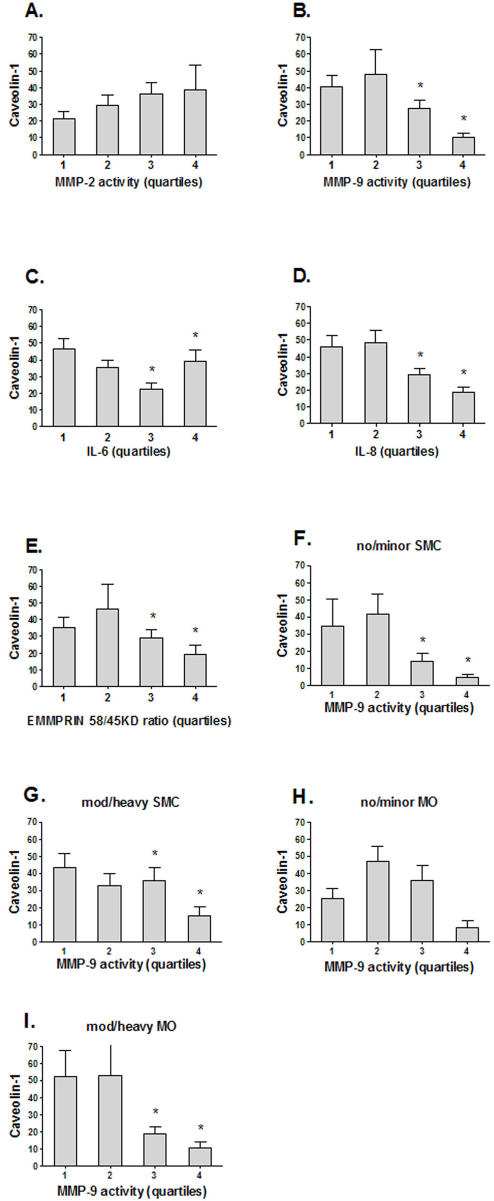
Relationships between Cav-1, Interleukin levels, MMP activity and EMMPRIN levels A, B, C, D: MMP-2, -9 and IL-6,-8 vs. Cav-1 protein expression levels. E: EMMPRIN 45/58KD ratio vs. Cav-1 protein expression levels. F, G: Analysis of association between MMP-9 and Cav-1 levels in plaques with no or minor smooth muscle cell (SMC) staining vs. plaques with moderate or heavy smooth muscle cell staining. H, I: Analysis of association between MMP-9 and Cav-1 levels in plaques with no or minor macrophage (MO) staining vs. plaques with moderate or heavy macrophage staining. Cav-1 levels are given as mean and standard error. P-values were calculated comparing quartiles 1 and 2 to quartiles 3 and 4 with the Mann-Whitney U test. * denotes p<0.05.

Next, we investigated the relationships between Cav-1 expression levels, local MMP activity, expression of the MMP-inducer CD147/EMMPRIN and the levels of pro-inflammatory cytokines (Figure 3). High MMP-9 activity was observed in patients with low Cav-1 expression levels (p<0.001) while there was no significant correlation between Cav-1 levels and MMP-2 (Figure 3A, B). Previously, we observed positive associations between MMP-9 expression and 58kD glycosylated EMMPRIN and MMP-2 and 45KD EMMPRIN levels, respectively [21]. Therefore, we studied the association between EMMPRIN glycosylation and Cav-1 expression. A significant association between Cav-1 expression and EMMPRIN glycosylation levels was observed (Figure 3E, p = 0.04). Low Cav-1 levels were associated with high IL-6 levels (p = 0.006) and IL-8 levels (p<0.001). These associations were not a mere reflection of constitutional expression of Cav-1 on certain cell types. As an example, Figure 3 (panel F-I) shows that the inverse associations between Cav-1 and MMP-9 are preserved within subgroups based on the number of smooth muscle cells and macrophages. Similar results were obtained for MMP-2, IL-6, IL-8, and EMMPRIN glycosylation (data not shown).

### Cav-1 Abundance, Clinical Presentation and Follow up

Having established the inverse association between local low levels of Cav-1 and the characteristics of a local vulnerable plaque phenotype, we investigated if local Cav-1 expression levels were related to clinical presentation at baseline and the occurrence of adverse events due to progression of atherosclerotic disease during follow up. Symptomatic patients presenting with transient ischemic attack or stroke had lower Cav-1 levels than asymptomatic patients but this difference did not reach statistical significance (Table 3; p = 0.13). There was no association between hypertension, diabetes, smoking and Cav-1 (Table 3). Women showed clearly higher levels compared with men (36.1 vs. 15.8; p<0.001). Patients with a history of myocardial infarction had lower levels of Cav-1 than patients with no such history (13.3 vs. 20.5; p = 0.04). Low levels of Cav-1 were found in patients with restenotic lesions: 6.1 vs. 19.7 (p = 0.04) (Table 3).

**Table 3:** Relations between clinical characteristics and Cav-1 expression levels.

Clinical characteristics	prevalence (%)	Cav-1 levels		p-value
		+	-	
age>70	43%	18.0 (5.5–44.0)	20.5 (6.7–49.6)	0.29
male	68%	15.8 (4.7–36.6)	36.1 (15.5–62.4)	<0.001
hypertension	70%	19.5 (5.2–45.9)	22.3 (7.3–48.2)	0.42
diabetes	20%	17.6 (7.7–49.6)	19.6 (5.5–45.6)	0.90
smoking	26%	18.6 (6.2–48.3)	19.9 (5.4–46.0)	0.92
BMI >30 kg/m^2^	14%	25.3 (5.5–50.2)	20.0 (6.7–47.1)	0.98
total cholesterol > 5.0 mmol/l	47%	22.9 (6.1–49.5)	19.8 (4.7–47.5)	0.55
HDL <1.2 mmol/l	38%	22.1 (4.9–50.2)	20.1 (5.7–41.1)	0.58
LDL > 3.0 mmol/l	40%	20.3 (6.6–55.9)	22.2 (4.8–44.9)	0.90
triglycerides > 2.0 mmol/l	39%	24.6 (6.2–25.6)	19.4 (5.2–44.1)	0.36
hs-CRP > 3.4 mg/l	49%	20.0 (5.3–49.6)	22.3 (5.2–49.5)	0.94
Restenotic lesion	3%	6.1 (0.7–28.1)	19.7 (6.9–48.0)	0.04
History of myocardial infarction	20%	13.3 (3.2–40.7)	20.5 (7.4–48.4)	0.04
History of angina pectoris	35%	17.2 (3.9–44.7)	20.5 (7.7–48.4)	0.21
Intermittent claudication	46%	18.1 (4.5–46.2)	20.5 (6.8–46.2)	0.46
***Medication***				
statin	64%	18.0 (4.6–46.0)	22.9 (8.0–46.2)	0.27
aspirin	84%	18.9 (5.4–48.6)	23.0 (10.2–45.7)	0.55
NSAID	6%	19.6 (9.8–53.7)	19.4 (5.7–46.0)	0.75
ACE inhibitor	43%	17.1 (4.8–45.3)	20.8 (7.2–49.3)	0.20
beta blocker	45%	19.8 (5.7–46.0)	18.3 (5.9–46.7)	0.62
***Clinical presentation***				0.13 *
asymptomatic	23%	23.6 (8.2–53.0)		
ocular symptoms	14%	27.1 (10.2–45.9)		
TIA	35%	15.3 (4.4–45.2)		
stroke	28%	17.1 (7.7–48.2)		

Median Cav-1 levels and interquartile range are given for patients in whom a clinical characteristic is present (+) or absent (−); e.g. age>70 (+) denotes the patient group older than 70 years.

* TIA and stroke compared with asymptomatic

**Figure 4:**
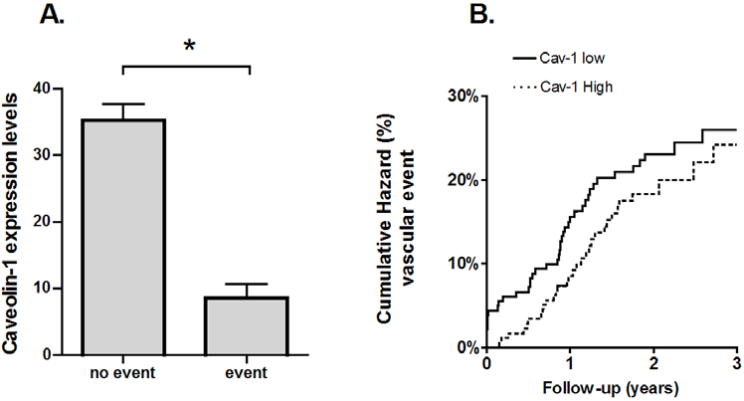
Caveolin-1 and Vascular Outcomes during Follow-up. A - Caveolin-1 expression levels in the plaque in relation the occurrence of an adverse vascular event within 30 days follow-up. *: p = 0.03. B - Cumulative hazard of vascular events during long-term follow-up. The solid line corresponds to patients with Cav-1 levels smaller than the median, and the dashed line corresponds to patients with Cav-1 levels larger than or equal to the median. Hazard ratio = 0.77 [0.48–1.23] (high vs. low Caveolin-1 expression)

Total follow-up included 625 patient years (mean 23 months) and 13 patients were lost to follow-up (3%). In total, 92 outcome events occurred (Table 4). Patients with vascular events within 30 days of surgery (8/378) had significantly lower Cav-1 levels than patients with no peri-operative events (Figure 4A; p = 0.03) and all of these patients had Cav-1 levels lower than the median Cav-1 level (p = 0.005). As shown in Figure 4B, this difference tended to persist during the first year after the intervention. However, no significant difference persisted during longer follow-up. At longer follow up,the Hazard Ratio (HR) for vascular events (Cav-1 > = median vs. Cav-1 <median) was 0.77 [0.48–1.23] (Figure 4B). The HR for cardiac events and ischemic strokes separately were similar: 0.76 [0.36–1.59] and 0.67 [0.22–2.03] respectively. Adjusting for age, sex and overall plaque phenotype did not markedly change these associations: HR for vascular events: 0.83 [0.51–1.36], HR for cardiac events 0.68 [0.31–1.48] and HR for ischemic stroke 0.85 [0.25–2.89].

**Table 4:** Occurrence of outcome events during follow-up.

	follow-up interval	
	30 days	total
person-years of follow-up*	31	625
vascular death	2	16
non-fatal ischemic stroke	2	11
non-fatal cerebral bleeding	1	1
non-fatal myocardial infarction	1	7
non-fatal ruptured aortic aneurysm	0	0
coronary revascularization	2	15
peripheral vascular intervention	0	42

* years of follow-up until occurrence of primary outcome event or end of follow-up period

### Cav-1 Abundance, Proteolytic Activity and Inflammation, *in vitro* and Animal Data

We further extended the study on the associations between Cav-1 and MMP expressions by analyzing the contribution of gelatinase activity to intimal hyperplasia in Cav-1 null mice upon femoral artery injury using peri-adventitial cuffs.

**Figure 5:**
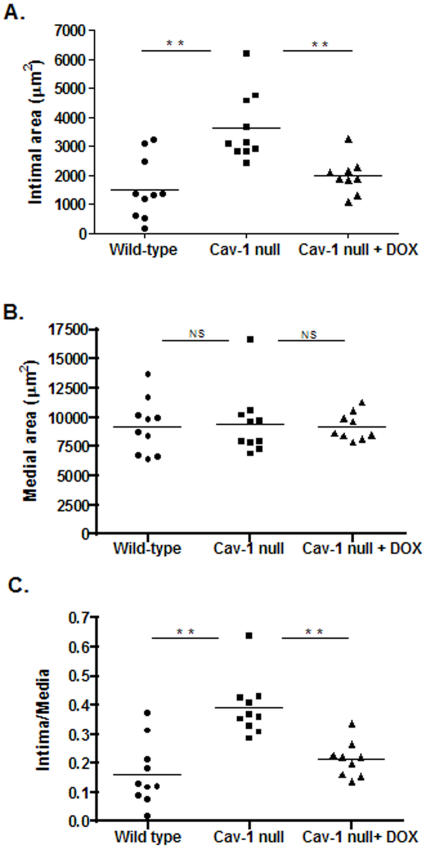
Morphometric analysis of cuffed femoral arteries. Total intimal area (A), medial area (B) and intima-media ratio (C) were quantified by image analysis using 6 serial sections in each cuffed artery. WT (n = 11) values are represented by circles, Cav-1 null (n = 10) are shown as squares and Cav-1 null+DOX (n = 9) are indicate as triangles, ** p<0.001.

Morphometric analysis revealed a significant increase in intimal area in Cav-1 null mice (WT = 1325±1069 µm^2^ vs Cav-1 null = 3627±1121 µm^2^ p = 0.003, Figure 5A). Medial area did not differ (WT = 9148±2340 µm^2^ vs Cav-1 null = 9449±2815 µm^2^ p = 0.4, Figure 5B) whereas intima-media ratio (WT = 0.12±0.1 vs Cav-1 null = 0.38±0.09 p = 0.001) was significantly larger in the Cav-1 null mice (Figure 5C).

Treatment with the MMP inhibitor doxycycline (DOX), significantly corrected the increased intimal hyperplasia response in Cav-1 null mice (Cav-1 null+DOX = 1975±620, Cav-1 null-DOX = 3627±1121 µm^2^ p<0.001, Figure 5A) while media area was not affected (Cav-1 null+DOX = 9217±1189 Cav-1 null-DOX = 9449±2815 µm^2^ p = 0.5, Figure 5B). Intima-media ratio also significantly reduced compared to untreated Cav-1 null mice (Cav-1 null+DOX = 0.21±0.06 Cav-1-DOX = 0.38±0.09 p<0.001, Figure 5C).

**Figure 6:**
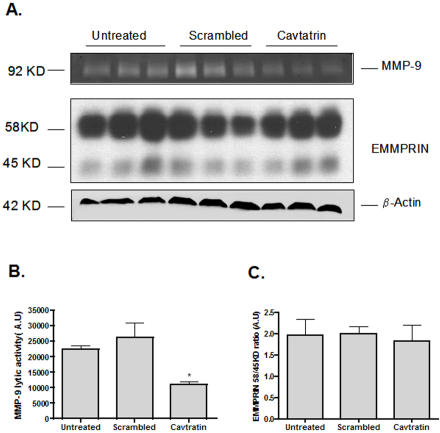
Cavtratin, MMP-9 and EMMPRIN in Raw-264.7. A- Top, representative zymogram of culture supernatant from Raw-264.7 macrophages. 1×10^6^ cells were incubated in a serum-free medium with Cavtratin or scrambled peptides (10 µM) for a period of 24 hours. Thereafter, culture media was collected and centrifuged for 5 minutes at 2000 rpm at 4C and subjected to gelatin zymography. Little or undetectable signal was observed for MMP-2. Middle, EMMPRIN expression levels in total cell lysates obtained from Raw-264.7 cells and representative western blot for β-actin (bottom panel). B- Quantification of MMP-9 lytic activity in the culture supernatant. * P = 0.02. C- Quantification of EMMPRIN expression levels. Data presented were normalized by β−actin expression.

To study if Cav-1 via its scaffolding domain (CSD) is involved in MMP regulation and the expression of pro-inflammatory mediators, we next evaluated the effect of cell-permeable synthetic peptides derived from the human CSD known as Cavtratin on gelatinolytic activity and cycloxygenase-2 (COX-2) expression in cultured murine Raw-264.7 macrophages. Peptides were efficiently taken up by the cells after 6 hours incubation (data not shown). Raw-264.7 cells were treated with 10 µM of Cavtratin or scrambled peptides for 24 hours and culture media was subjected to gelatin zymography. Cavtratin treatment significantly reduced MMP-9 levels (p = 0.02) after 24 hours of incubation (Figure 6A and B). The incubation of Raw-264.7 with the scrambled peptides did not affect MMP-9 levels in the culture supernatant or control β−Actin expression levels in the total lysate (Figure 6A and B). Very little, almost undetectable signal from MMP-2 was detected (data not shown). Since human carotid plaques showed an inverse relationship between Cav-1 expression levels and EMMPRIN glycosylation status, we additionally investigated whether the effect of Cavtratin on MMP-9 levels were mediated by changes in the glycosilation levels of EMMPRIN. As shown in Figure 6A and C, the ratios between low and higly-glycosylated EMMPRIN levels were not affected by any treatment (p = 0.7).

**Figure 7:**
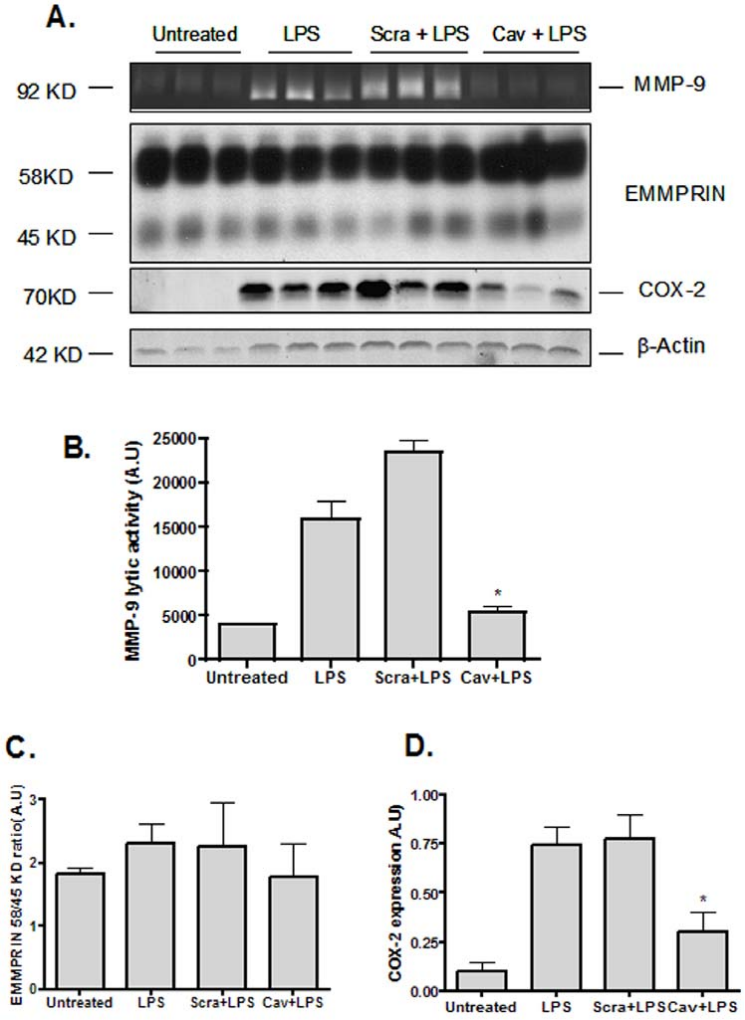
Cavtratin, MMP-9, COX-2 and EMMPRIN in LPS-challenged Raw-264.7. A- Top, MMP-9 activity in the culture supernatant from Raw-264.7 macrophages. 1×10^6^ cells were pre-incubated in a serum-free medium with Cavtratin or scrambled peptides (10 µM) for a period of 8 hours. Thereafter, LPS (10ng/mL) was added and the culture media was collected after 20 hours of LPS addition and centrifuged for 5 minutes at 2000 rpm at 4C and subjected to gelatin zymography. Middle top, representative western blot showing EMMPRIN expression in total cell lysates obtained from LPS-stimulated Raw-264.7 cells. Middle bottom, representative western blot for COX-2 expression in total cell lysates obtained from LPS-stimulated Raw-264.7 cells. Bottom panel, representative western blot showing β-actin expression. B- Quantification of MMP-9 lytic activity in the culture supernatant. * P<0.001. C- Quantification of EMMPRIN expression levels. Data presented were normalized by β−actin expression. D- Quantification of COX-2 expression levels. Data presented were normalized by β−actin expression. * P<0.001

To test if Cavtratin might prevent the MMP-9 activation and the induction of COX-2, Raw-264.7 cells were challenged with *E.coli* Lipopolysaccharide (LPS) in order to induce gelatinase activation and COX-2 expression. As expected, addition of LPS (10 ng/ml) for 20 hours, up-regulated total MMP-9 levels in the culture medium and COX-2 expression in the cell lysates (Figure 7). This up-regulation of active MMP-9 was significantly blocked by pre-incubation of cells with 10 µM Cavtratin (p = 0.01) (Figure 7 A and B). Induction of COX-2 expression after LPS stimulation was also markedly reduced by pre-incubation with Cavtratin (p<0.001). In contrast, pre-incubation of Raw-264.7 with scrambled peptides did not show any inhibitory effect either on MMP-9 or COX-2 levels (p = 0.4) (Figure 7 A, B and C). In all cases, no differences were found in β-Actin expression (Figure 7 A). We also determined EMMPRIN levels after LPS stimulation. As shown in Figure 7 A and C addition of LPS to Raw-264.7 did not have any impact on EMMPRIN expression levels. Pre-incubation of cells with either Cavtratin or scrambled peptides did not affect EMMPRIN levels (Figure 7 A and C).

**Figure 8:**
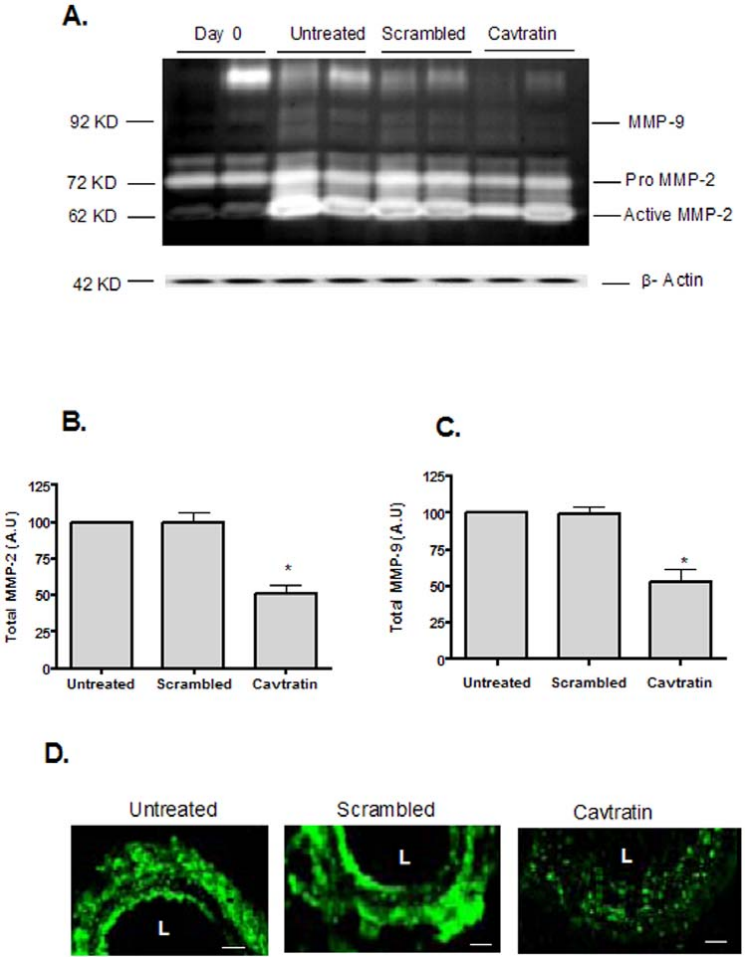
Cavtratin and Gelatinase Activity in Porcine Arterial rings. A- Representative zymogram of tissue homogenates from porcine arterial rings at baseline (0 days) and after 3 days in culture in the presence and/or absence of scrambled and Cavtratin peptides (10 µµ). Data represent 5 independent arterial rings., A representative western blot showing β-actin expression, demonstrating equal protein loading (bottom panel). B- Quantification of total MMP-2 (Pro and active MMP-2) in porcine arterial rings after 3 days in culture in the presence and/or absence of Cavtratin and scrambled (10 µµ). Data represent 5 different arterial rings. * p = 0.02 compared to untreated rings. C- Quantification of total MMP-9 in porcine arteries after 3 days in culture in the presence or absence of Cavtratin and scrambled (10 µM). Data represent 5 different arterial rings. * p = 0.01 compared to untreated rings. D- In situ zymography obtained from cutured rings. Gelatinolytic activity is shown in green. Scale bar = 100 µm. L = Lumen.

In parallel, this inhibitory effect of Cavtratin on gelatinolytic activity was also evaluated in arterial rings from porcine mammary artery that were cultured for 3 days with and without Cavtratin or scrambled peptide (10 µM). Gelatin zymography showed a significant down-regulation of the total lytic activity corresponding to MMP-2 (p = 0.02) and MMP-9 levels (p = 0.01) between Cavtratin and scrambled peptide treated or non-treated arteries (Figure 8 A, B and C). *In situ* zymography of the arteries suggests a reduction in total gelatinase activity in the presence of 10 µM of Cavtratin (Figure 8D) while treatment with scrambled peptides did not show any effect (Figure 8 D).

### Effect of Cav-1 Scaffolding Domain on Arterial Expansive Remodeling

**Figure 9:**
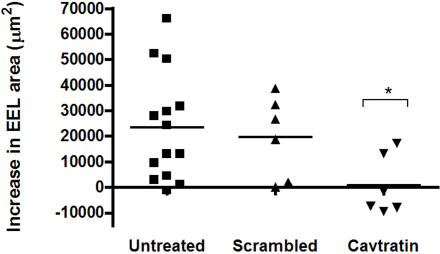
Cavtratin and Expansive Remodeling. Increase in EEL area (µm^2^) of the left carotid arteries (contralateral arteries) after ligation of the right carotid artery in BALB/c (n = 14) (circles), BALB/c+scrambled (1.5 mg/kg/day) (n = 6) (squares) and BALB/c+Cavtratin (1.5 mg/kg/day) (n = 6) (triangles). *p = 0.02

Since compelling evidence is pointing to arterial expansive remodeling as a major determinant of plaque vulnerability [28]; [29], we next questioned whether this process might be targeted by Cavtratin. For this, we evaluated the impact of Cavtratin administration on BALB/c mice by using the carotid artery contra-lateral to the ligation as a model in which only expansive remodeling takes place. Four weeks after ligation, the contra-lateral arteries showed an increase in total EEL area (23330±5644 µm^2^, *p* = 0.001 compared to non-ligated control arteries) (Figure 9). Mice treated with scrambled peptides (1.5mg/Kg/day) did not show any difference in EEL area increase compared to untreated arteries (19944±5434 µm^2^) (Figure 9). However, treatment of BALB/c mice with Cavtratin (1.5mg/Kg/day) resulted in a significant reduction in EEL area (735±4838 µm^2^, p = 0.02 compared to non-ligated control arteries) (Figure 9).

## Discussion

The present study identifies Cav-1 as a potential stabilizing factor in human atherosclerosis. Cav-1 levels were lower in atherosclerotic plaques compared to unaffected arteries and low Cav-1 levels were strongly associated with features of plaque vulnerability. Consistently, neo-intima formation after femoral cuff placement was increased in Cav-1 null mice, which could be reversed by addition of a MMP-inhibitor. Over-expression of the active domain of Cav-1 impaired inflammation, MMP-activity and arterial expansive remodeling. In addition to our descriptive clinical data, we show that patients with high plaque Cav-1 expression seem to be protected from cardiovascular events within 30 days after surgery, making Cav-1 the first available plaque biomarker with a prognostic value. The concept on local plaque markers that are predictive for adverse cardiovascular events that originate elsewhere in the vascular system is currently explored.

Several processes such as elevated proteolytic activity, inflammation, and expansive remodeling are directly related to plaque rupture [4]; [5]; [28]. Using different experimental approaches including cultured cells, animal models and the determination of Cav-1 levels in plaque specimens, we have evaluated the involvement of Cav-1 in the above-mentioned processes. We initially found that Cav-1 levels were inversely associated with MMP-9 activity and the glycosylation status of the MMP inducer, known as EMMPRIN, in carotid plaques. We next tested whether gelatinase activity contributes to enhanced intimal hyperplasia development in injured Cav-1 null femoral arteries. Here, we show that cuffed-arteries in Cav-1 null mice have larger intimal area and intima-media ratio than WT mice. These results are in line with a previous study reporting that carotid artery ligation in Cav-1 null resulted in an increased intimal area response [14]. Additionally, we studied the contribution of gelatinase to intima formation in Cav-1 null mice; MMPs were pharmacologically targeted using the MMP inhibitor doxycycline. Besides its anti-microbial actions, doxycycline is able to reduce expression and activity of several MMPs, including MMP-2 and MMP-9 [30]. MMP inhibition in Cav-1 null mice resulted in a significant attenuation of the increased intimal formation in Cav-1 null mice.

The existence of a positive relationship between Cav-1 and MMP-9 and the negative association with MMP-2 in the human specimens is supporting previous observations in which MMP-9 but not MMP-2 is associated with a stable plaque phenotype [21]. In a previous study, we demonstrated that MMP-2 is strongly associated with the presence of plaque stabilizing smooth muscle cells while MMP-9 is associated with the presence of inflammatory cells. Therefore, the negative association of Cav-1 with MMP-9 but not MMP-2 is supporting the hypothesis that Cav-1 is a plaque stabilizing molecule. As mentioned earlier, EMMPRIN glycosylation has previously been associated with either a stable [the 45KD glycosylated form] or an unstable plaque phenotype [the 58KD glycosylated form] [21]. Therefore, we considered that EMMPRIN glycosylation could be the mechanism by which MMP-9 expression is controlled via Cav-1. For this reason the expression of MMPs and EMMPRIN glycoslyation in relation to Cav-1 were studied in more depth.

Intracellular delivery of peptides that mimic Cav-1 scaffolding domain (CSD) (Cavtratin) in mouse macrophages and arterial rings impaired gelatinase activity. These results are in agreement with recently published data showing that Cav-1 inhibits MMP-2 activity in the heart and MMP-2 activity can be blocked using purified CSD [31]. Another potential mechanism by which Cav-1 might affect MMP cascade might imply changes in glycosylation status of EMMPRIN. It has been shown that up-regulation of Cav-1 prevents transition of EMMPRIN to a highly glycosylated (HG) form [32]; [33]. This HG-EMMPRIN forms a homodimer and augments MMP production and subsequent activation [32]; [33]. In our in vitro studies we could not demonstrate an effect of Cavtratin on EMMPRIN levels and therefore we could not prove that Cav-1 influences EMMPRIN glycosylation levels. Whether the reduction of Cav-1 levels leads to an increase in EMMPRIN 58kD glycosylation forms requires further investigation.

MMP-9 activity plays a major role in expansive remodeling in animal models [5]. Expansive remodeling is clinically associated with aortic aneurysms formation and a vulnerable atherosclerotic plaque phenotype [28]. Previously, it was shown that pharmacological inhibition of MMPs impaired arterial expansion upon vascular injury [34]. Therefore, we assessed the inhibitory potential of Cavtratin on this process. Here we show that treatment of mice with Cavtratin significantly reduced carotid expansive remodeling after flow increase. Although mechanically different, MMP-2 and MMP-9 are considered to play similar roles in atherosclerotic expansive remodeling and flow induced expansion of the artery. The current results therefore point to another potential mechanism by which Cav-1 may prevent plaque growth and destabilisation: expansively remodeled lesions may hide accelerated plaque growth with formation of large lipid pools with massive inflammatory response and protease activity that remains clinically silent and untreated for a long time.

We focused on protease activity as a mechanism by which Cav-1 exerts protective effects that would explain the observed outcomes in the clinical and animal studies. Although our results strongly suggest that a mechanistic link exists between Cav-1 and protease activity, we cannot rule out that Cav-1 has broader anti-inflammatory properties. The lower amount of IL-6 and IL-8 found in plaques with high Cav-1 content indeed suggests the importance of Cav-1 in the regulation of pro-inflammatory cascades associated with atherosclerosis. We also showed that incubation with Cavtratin reduced COX-2 expression in LPS-stimulated macrophages. Likewise, over-expression of Cav-1 full length in macrophages was capable of reducing inflammation via a MAPK-dependent mechanism [35].

The factors that are driving Cav-1 down-regulation during atherogenesis need further investigation. The clinical descriptive data obtained in this study may be helpful to generate hypothesis to answer these questions. Interestingly, we have found that women showed significantly higher Cav-1 expression levels than men. It is known that Cav-1 expression levels are up-regulated upon estrogen treatment in vascular smooth muscle cells [36]. Thus, this observation could suggest that Cav-1 is a potential player in gender associated differences observed in atherosclerotic disease [37]. Low expression levels of Cav-1 were observed in restenotic plaques, although restenotic plaques in general have a fibrous phenotype with high smooth muscle cell content. However, these plaques are in a highly proliferative status. Cav-1 is now recognized as an inhibitor of smooth muscle cell proliferation and decreased Cav-1 expression has been linked to increased smooth muscle cell proliferation in human atheroma [16]. Thus, the low levels of Cav-1 found in restenotic and unstable plaques could be related to a hyperproliferative cell phenotype.

Next to the descriptive clinical observations, we also found a relation between local Cav-1 expression and new peri-operative (30 days) vascular events. The ATHERO-EXPRESS is the first vascular bio-bank with a longitudinal design which allows studies on locally expressed biomarkers in the atherosclerotic plaque as surrogate marker to identify the so-called vulnerable patient [9]. This unique design allows investigating the process of atherosclerotic plaque progression independent of the traditionally identified vulnerable plaque characteristics as defined by post-mortem series. In the current study, all patients in the group with high Cav-1 levels (> = median level) did not suffer any adverse event in the peri-operative period. This suggests that down-regulation of Cav-1 levels in the vascular tree may induce a vulnerable status and atherothrombotic events, while patients with high Cav-1 levels seem protected. In addition, patients with a history of myocardial infarction had lower levels of Cav-1, supporting the relation between local plaque levels of Cav-1 and vulnerability for vascular events. A systemic predisposition to vascular events and plaque irregularity beyond classical cardiovascular risk factors has been shown on basis of carotid angiograms [38] . Since the systemic vulnerability ultimately leads to formation of an unstable plaque, it is conceivable that the atherosclerotic plaque hides information regarding systemic vulnerability. Although we observed a trend towards lower risk of vascular events in patients with high Cav-1 during follow-up after 30 days, the difference was not statistically significant.

In summary, our findings demonstrate a strong reduction of Cav-1 levels in human carotid plaques in comparison to non-atherosclerotic arteries. Low Cav-1 levels were associated with signs of plaque vulnerability and conversely, plaque Cav-1 levels showed a positive correlation with plaque stabilizing elements. Clinical follow-up revealed that high Cav-1 levels were associated with absence of major adverse cardiovascular events within 30 days of surgery.

Previous reports have shown other *in vivo* actions of Cavtratin targeting inflammation, tumor progression and right ventricle hypertrophy [24]; [25]; [39], We provide evidence showing that Cav-1 might act as a novel stabilizing factor in human atherosclerosis.

Although our data demonstrate that Cav-1 expression levels influence MMP activity within the plaques contributing to plaque instability, we can not rule out at this point other possibilities regarding how Cav-1 might influence plaque stability such as reduction in the total number of Caveolae and/or effects on plaque vascularisation.
